# Annotations

**Published:** 1894-05-26

**Authors:** 


### Annotations.

### A Provident Dispensary Failure.

What is there in medical men which prevents them combining for their own protection, and which almost universally makes their combinations failures when they do combine? Whatever it is, it is not to their credit. Dr. Alexander Stewart, of Manchester, has recently taken the Manchester Medico-Ethical Association into his confidence with regard to his experience of ten or twelve years in connection with a provident dispensary at Pendleton. So far as that particular provident dispensary is concerned, Dr. Stewart regretfully reports a practical failure. There must be some reasons for this. What are those reasons? The main reasons, Dr. Stewart thinks, may be reduced to two. The first is that the medical profession habitually gives so largely of its services to charitable and other institutions for nothing, that the public has come to think it has a right to claim those services for nothing, or for next to nothing. The second is that medical men can never be got to stand loyally by each other. If one doctor resigns an appointment because it is underpaid, or demands a serious sacrifice of self-respect, a dozen others rush in to fill the vacant place, regardless alike of poor pay or loss of self-respect. There is no beating about the bush here. The truth is plainly told, and everybody knows it to be the truth. For our part we should say that if large numbers of doctors have a preference for starvation and kicks, they must be allowed to indulge their preference. Our only wish would be that they should be well starved and kicked thoroughly. But it so happens that there are still a good many medical men left who respect themselves, and it is extremely desirable, in the interests of the public and of general morality, that all doctors should do so. It is necessary, therefore, to hope against hope, to teach the dull, and to persuade the blindly selfish against the excessive selfishness of professional fraticide or suicide. Provident dispensaries ought to be the doctor's natural bulwarks against a too aggressive out-patient system. They would be so if doctors could be got to fulfil the one condition of loyalty to each other. It should be a part of the serious business of every medical man's life to bring about this universal loyalty.

### Why is Suicide on the Increase?

It cannot any longer be denied that the number of suicides is rapidly increasing in this country as well as on the Continent. The *Spectator* gives two reasons for this. One chief cause, it affirms, is the "dwindling of religious faith." Another is the want of "elasticity of nerve." That is to say, in the region of mind people have lost hope, and in the region of the body they have lost pluck. No facts are given in support of these contentions, but there are few grounds for questioning their soundness, and many for admitting it. One or two facts, it seems to us, offer strong confirmation of the *Spectator's* contention. In Scotland, we believe, it will be found that as yet, at any rate, there is little or no increase in the number of suicides. Now in Scotland religious faith is still strong among the better as well as among the poorer classes, and physique and nerve are less impaired than in the more southern parts of the Kingdom. Indeed it is probable that in London itself will be found the principal part of the increase in the number of suicides. Now London has a poorer general physique and nerve, not only than Scotland, but also than other parts of the United Kingdom. Moreover, it is decidedly more inclined to Atheism, or at any rate to indifference in matters religious. Are these facts so? If they are, it is time the thoughtful among us were taking measures to check the moral disintegration which is beginning to prevail, and to consider also what steps, if any, can be taken to secure an improved physique for Londoners. One thing is certain, and that is that suicide shows feebleness both of mind and body. If the best our daily and weekly prophets, the newspapers, can do for mind and body is to weaken them, it might be well for us to consider the propriety of dropping our newspapers and doing something to revive the reputation and the flagging energies of the pulpit. Probably no class of human beings work harder or are more poorly paid than the adherents of the Salvation Army. But in the Salvation Army suicide is practically unknown. If it be the province of science to deal impartially with facts, here is a fact which is well worthy of consideration.

### The "Religio Medici."

A new edition of Sir Thomas Browne's "Religio Medici" has just been issued, by subscription, by Mr. G. Moreton, of Canterbury. The subscribers number over a thousand, and consist, we are glad to see, principally of medical men; though, as was to be expected; a good many clergymen and other lovers of literature have availed themselves of an opportunity, all too rare, to become the owners of so pleasing an English classic. A glance down the list of subscribers is full of interest to the medical reader who has been at pains to take the intellectual measure of the more outstanding of his professional contemporaries. The names of many men of light and leading are conspicuous by their absence. But they are by no means always the names of those doctors who might be supposed by a thoughtless public to have little regard for religion, either that of medicine or otherwise. Many people regard Professor Huxley, for example, as a sturdy opponent of religion. That, at any rate for a good many years, has not been our opinion. That learned man of science, like the late Mr. Darwin, sets, we feel sure, a very high value upon reasonable religion. His name, we are glad to note, figures among those of the subscribers to Mr. Moreton's reissue of the "Religio Medici." London does not furnish many names to the list of subscribers. The average London practitioner is not a literary person, we presume for want of time. The English provinces have furnished many well-known names; and Scotland is to the fore with most of her conspicuous men, notable among these being Dr. Clouston, of Edinburgh, and Professor W. T. Gairdner, of Glasgow. It is very satisfactory to find that one in thirty of all the members of the profession in the United Kingdom has become a possessor of the Norwich physician's venerable and world-famous work.

